# Evolution of elaborate parental care: phenotypic and genetic correlations between parent and offspring traits

**DOI:** 10.1093/beheco/arw129

**Published:** 2016-08-22

**Authors:** Clare P. Andrews, Loeske E. B. Kruuk, Per T. Smiseth

**Affiliations:** ^a^Institute of Evolutionary Biology, School of Biological Sciences, University of Edinburgh, Charlotte Auerbach Road, Edinburgh EH9 3FL, UK,; ^b^Institute of Neuroscience, Newcastle University, Framlington Place, Newcastle upon Tyne NE2 4HH, UK, and; ^c^Division of Ecology, Evolution and Genetics, Research School of Biology, The Australian National University, Canberra, ACT 2601, Australia

**Keywords:** heritability, multivariate analysis, *Nicrophorus vespilloides*, parental quality, trade-offs.

## Abstract

The evolution of elaborate forms of parental care is an important topic in behavioral ecology, yet the factors shaping the evolution of complex suites of parental and offspring traits are poorly understood. Here, we use a multivariate quantitative genetic approach to study phenotypic and genetic correlations between parental and offspring traits in the burying beetle *Nicrophorus vespilloides*. To this end, we recorded 2 prenatal traits (clutch size and egg size), 2 postnatal parental behaviors (direct care directed toward larvae and indirect care directed toward resource maintenance), 1 offspring behavior (begging), and 2 measures of breeding success (larval dispersal mass and number of dispersing larvae). Females breeding on larger carcasses provided less direct care but produced larger larvae than females breeding on smaller carcasses. Furthermore, there were positive phenotypic correlations between clutch size, direct, and indirect care. Both egg size and direct care were positively correlated with dispersal mass, whereas clutch size was negatively correlated with dispersal mass. Clutch size and number of dispersed larvae showed genetic variance both in terms of differences between populations of origin and significant heritabilities. However, we found no evidence of genetic variance underlying other parental or offspring traits. Our results suggest that correlations between suites of parental traits are driven by variation in individual quality rather than trade-offs, that some parental traits promote offspring growth while others increase the number of offspring produced, and that parental and offspring traits might respond slowly to selection due to low levels of additive genetic variance.

## Lay Summary

Although the evolution of parental care is a central topic in behavioral ecology, we know relatively little about how complex forms of care evolve. We studied elaborate parental care in the burying beetle *N. vespilloides*. Our results suggest that females differ in their parental quality, that some parental behaviors enhance offspring growth while others enhance offspring survival, and that there is little genetic variation in parental and offspring behaviors.

Twitter: @persmiseth

## INTRODUCTION

Parental care is an integral part of reproduction in a wide range of animals, and occurs whenever parents enhance the survival and growth of their offspring, often at a cost to their own survival and reproduction (Royle et al. 2012). Understanding the evolution of parental care is an important aim in behavioral ecology, given that there is a great amount of diversity in its form, level, and duration (Clutton-Brock 1991; Royle et al. 2012). Traditionally, behavioral ecologists have considered the evolution of parental care in light of variation in its costs to the parents and benefits to the offspring (Clutton-Brock 1991; Royle et al. 2012). Although this approach is both general and tractable, it treats parental care as a simple unitary trait, whereas in reality parental care often involves complex suites of associated parental and offspring traits (Gardner and Smiseth 2011). For example, in most mammals and birds, as well as some insects, one or both parents supply nourishment first to the developing embryos via a placenta or in the form of egg yolk, and then as milk or other sources of food to the offspring after hatching or birth (Smiseth et al. 2012). Furthermore, offspring often influence parental care by soliciting food from their parents (Godfray 1991; Kilner and Johnstone 1997). Thus, there is now a need for studies that help advance our understanding of the conditions shaping the evolution of complex suites of parental and offspring traits.

Useful insights into the evolution of elaborate forms of parental care can be gained by examining the sources of variation and covariation determining associations between different parental traits (Walling et al. 2008), as well as between offspring and parental traits (Kölliker et al. 2000; Lock et al. 2004). The extent to which differences in parental and offspring traits are influenced by environmental and genetic variation determines their potential to respond to selection. Studies of correlations between suites of parental traits can provide evidence for trade-offs if such correlations are negative (Heaney and Monaghan 1996; Walling et al. 2008), or evidence for variation in resource acquisition (or “parental quality”) if such correlations are positive (van Noordwijk and de Jong 1986). Meanwhile, associations between parental and offspring behaviors can provide evidence for parent–offspring communication, if offspring begging influences parental care decisions or vice versa (Kilner and Johnstone 1997; Smiseth and Moore 2002). Further insights can be gained from quantifying genetic correlations between traits. For example, negative genetic correlations between parental traits suggest genetic constraints on the evolution of parental traits (Walling et al. 2008). Finally, genetic correlations between parental food provisioning and offspring begging provide evidence of coadaptation between parents and their offspring (Kölliker et al. 2000; Agrawal et al. 2001; Hager and Johnstone 2003; Lock et al. 2004; Maestripieri 2004; Lucass et al. 2016).

Until now, associations between different parental traits and between parental and offspring traits have been studied in a somewhat piecemeal fashion, focusing either on associations between pairs of postnatal parental traits (Walling et al. 2008) or on associations between offspring begging and parental provisioning (Lock et al. 2004; Lucass et al. 2016). However, we are lacking comprehensive analyses of phenotypic and genetic correlations between a wider range of parental care traits and between those traits and offspring traits, such as begging, that influence parental care decisions (Kilner and Johnstone 1997). Furthermore, we need analyses of how variation in parental and offspring traits contribute toward subsequent breeding success. Here, we address these gaps using a multivariate quantitative genetic approach to study phenotypic and genetic correlations between a set of parental and offspring traits in the burying beetle *Nicrophorus vespilloides*.

*Nicrophorus vespilloides* is ideal for examining the evolution of elaborate parental care for several reasons. First, care comprises a range of prenatal and postnatal parental traits that enhance larval growth and survival (Eggert et al. 1998; Smiseth and Moore 2002; Monteith et al. 2012), and larvae beg for food from parents (Smiseth and Moore 2002; Smiseth et al. 2003). Previous work on this species has documented negative phenotypic and genetic correlations between components of postnatal parental care (Walling et al. 2008), and positive phenotypic and genetic correlations between larval begging and parental food provisioning (Smiseth and Moore 2002; Lock et al. 2004). Second, *N. vespilloides* can be reared in relatively large numbers under naturalistic laboratory conditions, making it possible to obtain estimates of genetic variation underlying phenotypic traits through standard breeding designs (Lock et al. 2004; Walling et al. 2008). Finally, it is possible to manipulate the environmental conditions influencing parental and offspring traits to examine whether parents provide more or less care when conditions are favorable. For example, parents might be expected to provide more care if the costs of care are lower under favorable conditions. Alternatively, parents might be expected to provide less care if the care has a smaller effect on offspring fitness under favorable conditions. In this species, a key environmental variable is the amount of resources that parents have available for breeding, which can be readily altered by adjusting the size of the mouse carcass provided as a breeding resource (Smiseth et al. 2014).

The main aims of this study were therefore as follows: 1) to test for effects of variation in environmental conditions on a suite of parental and offspring traits; 2) to estimate phenotypic correlations (and covariances) between parental and offspring traits; 3) to estimate correlations between parental and offspring traits and measures of breeding success; and 4) to estimate heritabilities of, and genetic correlations (and covariances) between, parental and offspring traits. Addressing these aims will advance our understanding of the evolutionary processes shaping elaborate forms of parental care by providing valuable insight into potential trade-offs between different parental traits, communication between parents and offspring, and potential genetic constraints on the evolution of parental and offspring traits.

## METHODS

### Study species

*Nicrophorus vespilloides* breeds on carcasses of small vertebrates that they bury underground (Eggert and Müller 1997; Scott 1998). Females lay eggs in the soil surrounding the carcass. The eggs hatch asynchronously and the newly hatched larvae then crawl to the carcass (Smiseth et al. 2006). One or both parents provide prenatal care for the larvae by burying, defending and preparing the carcass (i.e., removing fur or feathers), and creating a opening termed a “crater” on the carcass within which larvae assemble to self-feed (Eggert and Müller 1997; Scott 1998). After larval hatching, parents provide elaborate postnatal care, which involves multiple parental behaviors such as cleaning the carcass of bacterial and fungal growth (Eggert et al. 1998; Rozen et al. 2008) and regurgitating predigested carrion to the larvae, which enhances growth and speeds up development (Smiseth et al. 2003; Lock et al. 2004). Larval behaviors include begging for food from their parents, and self-feeding directly from the carcass (Smiseth et al. 2003). The larvae stay on the carcass for 5–7 days, during which time they achieve a 90-fold increase in body mass (Smiseth et al. 2003).

### Origin of beetles and animal husbandry

We conducted our experiments on a large outbred laboratory population maintained at the University of Edinburgh. This population was derived from beetles that had been caught in the wild at 2 locations several generations back (see below for further details): Corstorphine Hill, Edinburgh, and Kennall Vale, Cornwall (both United Kingdom). The laboratory population was kept under 24-h light and at 20°C following established protocols (Smiseth and Moore 2004). Each generation, we bred pairs of nonsibling virgin females and males from the same population of origin (i.e., Edinburgh or Cornwall). Beetles selected for breeding were placed in transparent plastic boxes (12×10×2.5cm) containing about 1cm moist soil and a previously frozen mouse carcass (Livefoods Direct Ltd, Sheffield, UK). When larvae dispersed from the carcass, we placed them into individual transparent plastic containers (12×8 × 2cm) with 1cm moist soil to pupate. Following eclosion, all adults were fed pieces of organic beef twice a week.

### Breeding design

We used beetles from generations 2–6 from the Edinburgh-derived population and from generations 7–13 from the Cornwall-derived population, which were bred in a full-sib, paternal half-sib mating design (e.g., Fig. 18.3 in Lynch and Walsh 1998). In the first generation of the breeding experiment, we selected 78 unrelated virgin sires, each of which was paired with 3 or 4 randomly selected unrelated virgin dams. We only paired males with a fourth female if one (or more) of the first 3 pairings failed to produce any offspring. The male was left to mate with each female for 3–4 days on a mouse carcass of a standardized size (mean ± standard error: 18.7±0.2g). Given that some matings failed to produce offspring, we had a total of 192 broods, each comprising 1–48 larvae, in the first generation of the experiment.

In the second generation of the breeding experiment, we randomly selected 6 full-sib sisters from each full-sib family (or fewer if only a smaller number of females were alive) derived from the first generation of mating. To examine effects of variation in environmental conditions on parental and offspring traits, we manipulated the amount of resources available for breeding by providing females with different-sized mouse carcasses. For each set of 6 full-sib sisters, 3 were provided with a small carcass (10–15g) and 3 were provided with a large carcass (20–25g). Each of these females was mated to a different unrelated male also derived from the first generation of the experiment. For each female, we collected information on parental and offspring traits as well as breeding success (see further details below). In total, we collected measurements on parental and offspring traits and breeding success from 465 pairs. For practical reasons, we conducted the experiment in 6 blocks (hereafter termed “batches”) over a period of over 10 months with 58–146 broods per block. Because the experiment ran over several months, some of the first generation parents used in the later batches were descendants of parents from the earlier batches, resulting in a multigenerational pedigree with a total of 781 sires and 866 dams.

In all second-generation matings, we removed the male 48h after pairing. We did this because male involvement with parental care is highly variable and because male assistance in provisioning has no detectable effect on larval growth or survival under laboratory conditions (Eggert et al. 1998; Smiseth et al. 2005). Thus, male removal excludes potential confounding effects due to the highly variable amount of care provided by males (Smiseth et al. 2005).

### Trait measurements

For each brood, we collected data on 7 traits as follows: 1) clutch size, 2) egg size (batches 3–6 only), 3) direct care, 4) indirect care, 5) time spent begging, 6) dispersal mass, and 7) number of dispersed larvae. Clutch size and egg size were used as indicators of *prenatal parental investment*, whereas direct care and indirect care were used as indicators of *postnatal parental care*. We included time spent begging in the analyses because this is an *offspring behavior* that is known to influence parental care decisions (Smiseth and Moore 2002). Finally, number of dispersed larvae and dispersal mass were included as measures of *breeding success*.

#### Prenatal parental investment

We measured *clutch size* by counting the number of eggs that were visible from the underside of the container 48h after pairing. To verify that this count provided a reliable estimate of clutch size, we compared counts based on number of eggs visible from the underside of the container and counts made by emptying the box and manually sifting through the soil. There was a strong positive correlation between the 2 counts (*r*_s_ = 0.98, *n* = 21, *P* < 0.001), confirming that the former count provided a reliable estimate of clutch size. To measure *egg size*, we photographed the bottom of the transparent boxes from below, including a scale bar. We then measured the length and width of a sample of 6 randomly selected eggs per brood, using ImageJ (Abramoff et al. 2004). Following existing protocols, we used these measures to calculate the volume *V* for each egg using the equation *V* = (1/6)π*w* ^2^ *L*, where *w* is the width and *L* the length of the egg (Berrigan 1991; Monteith et al. 2012). We then calculated an average egg volume for each female as the estimate of egg size.

#### Postnatal parental care

We recorded female parental behavior and offspring begging on the day following the hatching of the first eggs. To identify the time at which the first eggs in a brood started hatching, we checked containers for hatching 3 times per day from the morning of day 3 after pairing. We recorded the time when the first larvae were observed either in the soil or on the carcass. We did the observations on the following day when the eldest larvae in the brood were approximately 24-h old to coincide with the peak in parental care and larval begging (Smiseth et al. 2003). We recorded female care and offspring begging behavior following established protocols, using instantaneous sampling every 1min over a period of 30min (Smiseth and Moore 2002; Smiseth et al. 2003; Lock et al. 2004; Andrews and Smiseth 2013). For each 30-min observation period, we recorded the number of times the female was observed providing *direct care*, defined as when she was providing food to the larvae (engaging in mouth-to-mouth contact with at least 1 larva), interacting with the larvae (standing still within the crater and allowing the larvae to beg), or consuming carrion (feeding from within the crater), or *indirect care*, defined as when she was guarding (standing still in a position nearby the crater where she could defend the brood from predators and conspecifics) or maintaining the carcass (depositing anal or oral secretions to the carcass, manipulating the carcass from below, or excavating the crypt (i.e., the depression in the soil around the carcass (see Walling et al. 2008)). Our definition of direct care follows that used by Walling et al. (2008) rather than Mattey and Smiseth (2015). The reason for this is that we focused on female parents only, and a recent study suggests that female burying beetles regurgitate most of the carrion they consume to the larvae (Pilakouta et al. 2016). In contrast, male parents regurgitate far less of the carrion they consume to the larvae (Pilakouta et al. 2016), and consuming carrion should therefore be excluded from direct care in studies involving males (Mattey and Smiseth 2015). Indirect care was log-transformed to improve normality of residuals. All other behaviors were recorded as nonparental, including the female sitting, self-grooming, locomotion without performing any of the above behaviors, or absence from the crypt, and were excluded from further analyses.

#### Offspring behavior

At each scan, we recorded the number of larvae that were begging at that time point. We considered a larva to be begging when raising its head toward the parent (Rauter and Moore 1999). Because larvae beg only when a parent is close, we noted the number of scans in which the female and larvae were in close proximity (*P*), defined as a distance of less than the female’s pronotum width, corresponding to the approximate distance from the parent at which the larvae start begging (Rauter and Moore 1999; Smiseth and Moore 2002). Following observations, we counted the number of larvae (*L*) in each brood. We calculated the average percentage time spent begging by each larva in the brood in the proximity of the female as *b* = (Σ*b*/*L*) (100/*P*), where Σ*b* is the total number of begging and feeding events and *L* and *P* are defined as above (Smiseth and Moore 2004).

#### Breeding success

From day 8 onward, we checked broods daily for larval dispersal, defined as all larvae having left the crypt surrounding the remains of the carcass. We recorded the *number of dispersed larvae* by counting the number of larvae in the brood at the time of dispersal. We weighed the mass of the total brood (to within 0.1mg) and used this to calculate the average larval *dispersal mass* as brood mass divided by number of larvae. Larvae do not feed once they disperse from the carcass, and body mass at dispersal is therefore an accurate predictor of adult body size and mass, both of which are strongly related to survival and reproductive success as an adult (Otronen 1988; Lock et al. 2004).

### Statistical analyses

All statistical analyses were performed in ASReml-R version 3 (Butler et al. 2009). We first fitted multiple linear regression models for each trait to assess effects of carcass size, population of origin (i.e., Cornwall or Edinburgh), and batch (as a multilevel factor). We note that not correcting for population of origin and batch effects resulted in much higher estimates of heritability for all traits (see below). Based on the pedigree, we found that a small proportion of females had nonzero inbreeding coefficients (13% with *f* ≥ 0.03125). However, we found no evidence of inbreeding depression (i.e., no effect of including the inbreeding coefficient *f* as a fixed effect in our models) and we therefore excluded inbreeding coefficients from further analyses.

We next estimated phenotypic covariances and correlations between the 7 traits using a multivariate model in ASReml-R (Butler et al. 2009). To avoid scaling issues in the multivariate analyses, the values for all traits (including the log-transformed values for indirect care) were transformed to unit variance prior to use in the multivariate analyses. We did not centre the data on zero given that our aim was to estimate variance and variance components, which will not be affected by any centring. The statistical significance of each covariance component was assessed by comparison of the log likelihood with a model in which that covariance was constrained to zero.

We then used the pedigree to fit an “animal model” (Lynch and Walsh 1998; Kruuk 2004; Wilson et al. 2010) by adding an individual additive genetic random effect for each trait to the multivariate model to give estimates of the additive genetic variance (*V*_a_) for each trait, as well as the additive genetic covariances between traits. For the traits measured on female parents (clutch size, egg size, direct care, indirect care), we considered the additive genetic effect of each female, the covariances of which are defined by the pedigree structure. Dealing with the traits measured in offspring (dispersal mass, time spent begging, number of dispersed larvae) was more complex, as these traits were measured at the level of the brood rather than individual offspring. We initially treated the brood as a single offspring, and fitted additive genetic effects for each brood as defined by its parents. However, we found no evidence of significant additive genetic variance in any trait this way. For consistency with the parental traits, we therefore considered the offspring traits as if defined by the additive genetic effects of the female, which is effectively fitting a maternal genetic effect on the brood (Kruuk and Hadfield 2007). Estimates of additive genetic variance are therefore at the level of the female parent for all traits. These values were very low for some traits, so we were not able to estimate genetic covariances between all possible pairwise combinations. Statistical significance of the estimates of additive genetic variance was estimated by a likelihood ratio test comparing univariate models with and without additive genetic variance for each trait, and for covariances by a likelihood ratio test setting the respective covariance to zero in the full multivariate model.

We also tested for any effect of fitting the identity of the mother of each focal female as an additional random effect (i.e., testing for maternal effects on their daughters’ performance as parents), but as these were never significant, we excluded them from the final analyses. Finally, we considered genotype by environment interactions by adding an interaction between carcass size (small vs. large) and the estimate of additive genetic variance *V*_a_ in each model, and used a likelihood ratio test (with 2degrees of freedom) to test whether this was an improvement on the model with a single estimate of *V*_a_. For no trait did this result in a significant improvement to the model, and we therefore do not report these results in any further detail. Model residuals were inspected for normality and homogeneity. Given that the analyses involved multiple traits, we used *P* < 0.01 as significance level.

## RESULTS

### Environmental conditions and population of origin

All traits showed substantial phenotypic variation, with the highest coefficients of variation for indirect care and number of dispersed larvae (Table 1). Most traits were influenced by variation in either environmental conditions or population of origin. First, variation in carcass size (i.e., resource availability) had significant effects both on parental traits and on breeding success. Females breeding on larger carcasses provided significantly less direct care than females breeding on smaller carcasses (*P* < 0.001, Table 1), but the former still produced larvae with a higher average dispersal mass than the latter (*P* < 0.001, Table 1). Second, population of origin had significant effects on clutch size and number of dispersed larvae: females from the Cornwall-derived population produced both a larger clutch size and a larger number of dispersed larvae than females from the Edinburgh-derived population (Table 1). There was also a marginally nonsignificant (*P* = 0.015) reduction in direct care from the Edinburgh-derived population. Finally, there were significant differences between batches for clutch size, egg size, direct care, dispersal mass, and number of dispersed larvae (Table 1). In all following models, we correct for effects due to carcass size, population of origin, and batch for all traits.

**Table 1:** Summary statistics and effects of carcass size, population of origin, and batch differences from univariate models of each trait.

Trait	*N*	Mean	SD	CV	Carcass (SE)	Carcass *P*	Origin (SE)	Origin *P*	Batch *P*
Clutch size (count)	465	23.445	11.879	0.507	0.021 (0.099)	0.868	−10.417 (1.731)	<0.001**	<0.001**
Egg size (mm^3^)	217	2.738	0.377	0.138	−0.002 (0.005)	0.427			<0.001**
Direct care (% time)	362	9.865	9.485	0.961	−0.323 (0.091)	<0.001**	−6.548 (1.623)	0.015	<0.001**
Indirect care (% time)	362	3.486	3.956	1.135	−0.040 (0.039)	0.298	0.287 (0.704)	0.125	0.013
Time begging (% time)	250	6.839	4.077	0.596	0.004 (0.051)	0.836	0.029 (0.938)	0.511	0.130
Dispersal mass (mg)	342	167.87	30.60	0.182	1.940 (0.290)	<0.001**	−7.150 (5.540)	0.520	<0.001**
No. dispersed (count)	594	8.244	9.447	1.146	0.117 (0.069)	0.064	−10.319 (1.042)	<0.001**	<0.001**

The table shows sample size N, mean, SD, and coefficient of variation CV (SD/mean) for each trait. Effects of carcass size are measured in g (with SE), population of origin refers to Edinburgh-derived relative to Cornwall-derived population, and batch differences refers to 6 experimental blocks (see Methods for details). Sample sizes refer to the number females for which these traits were measured. Estimates for effects of population of origin on egg size are missing because egg size was measured for the Cornish population only. Significance is given by **P < 0.001. CV, coefficient of variation; SD, standard deviation; SE, standard error.

### Phenotypic correlations

**Figure 1:**
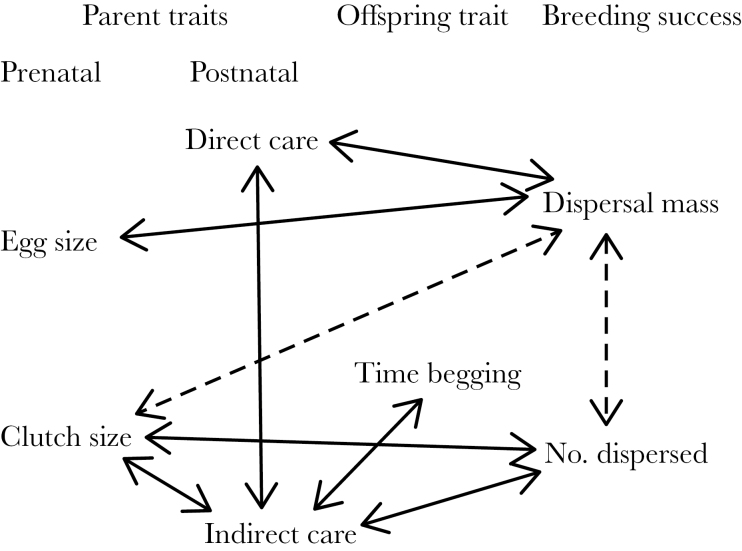
Summary of phenotypic correlations between prenatal parental investment (clutch size and egg size), postnatal parental care (direct and indirect care), offspring behavior (larval begging), and breeding success (dispersal mass and number of dispersing larvae). Significant negative correlations are shown in dashed lines, while significant positive correlations are shown in solid lines.

Table 2 and Figure 1 provide an overview of the phenotypic correlations and covariances between different traits. First, we examined phenotypic correlations between the 4 parental traits (i.e., clutch size, egg size, direct care, and indirect care). There was no significant correlation between egg size and clutch size (Table 2), suggesting no evidence for any trade-off between the number and size of eggs. However, as would be expected, if there were variation in parental quality, there was a significant positive correlation between direct and indirect care, and between clutch size and indirect care (Table 2). Second, we examined phenotypic correlations between parental traits and larval behavior (time begging). There was no correlation between time spent begging and direct care as would have been predicted if parents respond to begging by adjusting food provisioning. Instead, there was a significant positive correlation between time spent begging and indirect care (Table 2). Finally, we examined phenotypic correlations between the parental and offspring traits and our 2 measures of breeding success (i.e., dispersal mass and number of dispersed larvae). The number of dispersed larvae was positively correlated with clutch size and indirect care, whereas dispersal mass was positively correlated with egg size and direct care but negatively correlated with clutch size (Table 2). There was a negative correlation between the 2 measures of breeding success (Table 2), suggesting a trade-off between size and number of offspring.

**Table 2:** Multivariate phenotypic models with carcass size, population of origin and batch effects (see Methods) as fixed effects

	Clutch size	Egg size	Direct care	Indirect care	Time begging	Dispersal mass	No. dispersed
Clutch size	*0.830 (0.053*)	0.104 (0.069)	0.125 (0.053)	0.231 (0.051)**	−0.087 (0.066)	−0.184 (0.054)	0.635 (0.025)**
Egg size	0.085 (0.059)	*0.888 (0.085*)	0.046 (0.080)	−0.088 (0.075)	−0.032 (0.095)	0.219 (0.075)*	0.116 (0.066)
Direct care	0.109 (0.047)	0.038 (0.072)	*0.902 (0.068*)	0.166 (0.050)*	0.089 (0.064)	0.166 (0.053)*	0.099 (0.054)
Indirect care	0.203 (0.048)**	−0.089 (0.071)	0.161 (0.050)*	*0.954 (0.072*)	0.194 (0.063)*	0.036 (0.055)	0.326 (0.046)**
Time begging	−0.066 (0.063)	−0.036 (0.094)	0.114 (0.065)	0.232 (0.070)*	*1.019 (0.094*)	0.103 (0.065)	−0.042 (0.066)
Dispersal mass	−0.140 (0.046)	0.185 (0.067) *	0.145 (0.048)*	0.040 (0.049)	0.106 (0.062)	*0.835 (0.065*)	−0.191 (0.055)
No. dispersed	0.510 (0.042)**	0.095 (0.056)	0.087 (0.047)	0.291 (0.048)**	−0.020 (0.062)	−0.143 (0.047)*	*0.822 (0.048*)

The table shows the variance–covariance-correlation matrix, with phenotypic variances on the diagonal (italic cells), phenotypic covariances below diagonal, and phenotypic correlations above. Each value is followed by SE in brackets. Significance is given by *P < 0.01; **P < 0.001. Trait values were standardized to unit variance prior to analyses (see Methods). SE, standard error.

### Heritabilities and genetic correlations

Table 3 provides information on heritability estimates based on univariate models where we either controlled for effects due to population of origin and batch or, for comparison, did not control for such confounding effects. We found significant additive genetic variances in clutch size and number of dispersed larvae when we controlled for effects of population of origin and batch (Table 3). For both traits, the heritabilities were relatively low (0.16 and 0.20, respectively). There was no significant additive genetic variance for the remaining 5 traits (Table 3). If we did not control for possible effects of population of origin and batch, estimates of additive genetic variance were significant for 6 out of 7 traits (Table 3).

**Table 3:** Estimates of heritability from univariate models, both with and without correcting for population of origin and batch effects

	Correcting for origin and batch effects			Not correcting for origin and batch effects		
Trait	Heritability	SE	*P*	Heritability	SE	*P*
Clutch size	0.204	0.093	0.004*	0.465	0.107	<0.001**
Egg size	0.127	0.130	0.262	0.325	0.142	<0.001**
Direct care	0.012	0.067	0.854	0.156	0.085	0.008*
Indirect care	0.083	0.089	0.308	0.209	0.097	0.002*
Time begging	0.200	0.129	0.039	0.214	0.125	0.025
Dispersal mass	0.179	0.112	0.051	0.338	0.125	<0.001**
No. dispersed	0.156	0.068	0.001*	0.417	0.088	<0.001**

Significance is given by *P < 0.01; **P < 0.001, estimated from a likelihood ratio test comparison with a model in which additive genetic variance is not fitted. SE, standard error.

Table 4 provides information on the patterns of genetic covariances and correlations between the different traits based on a multivariate model. The only significant genetic correlation was between clutch size and number of dispersing larvae, which are also the 2 traits that had the highest heritabilities.

**Table 4:** Multivariate animal model with carcass size, population of origin and batch effects as fixed effects, and estimates of the additive genetic variance (*V*_a_) of the female

Residual (co)variances	Clutch size	Egg size	Direct care	Indirect care	Time begging	Dispersal mass	No. dispersed
Clutch size	*0.641 (0.074*)	−0.013 (0.110)	0.127 (0.060)	0.243 (0.058)**	−0.157 (0.104)	−0.202 (0.082)	0.595 (0.045)**
Egg size	−0.009 (0.079)	*0.807 (0.121*)	0.044 (0.084)	−0.113 (0.082)	−0.065 (0.112)	0.222 (0.082)	0.088 (0.094)
Direct care	0.097 (0.046)	0.038 (0.072)	*0.898 (0.085*)	0.178 (0.053)*	0.122 (0.073)	0.174 (0.056)*	0.099 (0.057)
Indirect care	0.185 (0.047)**	−0.097 (0.071)	0.160 (0.050)*	*0.907 (0.093*)	0.267 (0.074)*	0.054 (0.059)	0.343 (0.053)**
Time begging	−0.115 (0.076)	−0.053 (0.092)	0.105 (0.064)	0.232 (0.068)*	*0.831 (0.128*)	0.089 (0.099)	−0.092 (0.094)
Dispersal mass	−0.140 (0.060)	0.173 (0.067)	0.144 (0.048)*	0.045 (0.049)	0.071 (0.080)	*0.755 (0.088*)	−0.145 (0.075)
No. dispersed	0.398 (0.055)**	0.067 (0.072)	0.078 (0.046)	0.273 (0.047)**	−0.070 (0.071)	−0.105 (0.056)	*0.701 (0.060*)
Additive genetic (co) variances	Clutch size	Egg size	Direct care	Indirect care	Time begging	Dispersal mass	No. dispersed
Clutch size	*0.211 (0.084*)*	0.762 (0.543)			0.345 (0.357)	−0.023 (0.438)	0.770 (0.500)*
Egg size	0.105 (0.079)	*0.091 (0.108*)					0.390 (0.569)
Direct care			*0.003 (0.055*)				
Indirect care				*0.047 (0.070*)			
Time begging	0.070 (0.073)				*0.196 (0.128*)	0.355 (0.517)	0.434 (0.378)
Dispersal mass	−0.003 (0.057)				0.044 (0.069)	*0.079 (0.077*)	−0.346 (0.450)
No. dispersed	0.125 (0.058)*	0.041 (0.064)			0.068 (0.061)	−0.034 (0.047)	*0.125 (0.056*)*

The tables show the variance–covariance-correlation matrix, with residual and additive genetic variances on the diagonal (italic cells), covariances below diagonal, and correlations above. Each value is followed by SE in brackets. Blank cells indicate covariances not estimable, because of small variance components. Significance of covariances and correlations is given by *P < 0.01; **P < 0.001. Trait values were standardized to unit variance prior to analyses (see Methods). SE, standard error.

## DISCUSSION

### Environmental conditions and population of origin

Overall, we found evidence that environmental conditions affected variation in both parental care and breeding success in the burying beetle *N. vespilloides*. Such effects are not surprising given that the carcass is the sole source of food for the larvae and that its size determines the amount of resources available to the offspring (Smiseth and Moore 2002; Smiseth et al. 2014). The results also add to previous studies on *N. vespilloides*, and other species within the genus *Nicrophorus*, which have reported effects of carcass size on breeding success (Wilson and Fudge 1984; Bartlett and Ashworth 1988; Scott and Traniello 1990; Trumbo 1990; Smiseth and Moore 2002; Smiseth et al. 2014). However, a previous study using a wider range of carcass sizes reported stronger and more consistent effects of carcass size on the *number* of offspring produced than on offspring *size* (Smiseth et al. 2014). The lack of an effect on the number of dispersed larvae in our study may reflect differences in experimental design, although we note that there was a nonsignificant trend in the predicted direction in our data set (Table 1).

We found that females provided less direct care when they were breeding on larger carcasses. We are unaware of any previous evidence that carcass size influences parental behaviors despite that some previous studies on this species have tested for such effects (Smiseth and Moore 2002, 2004). It is noteworthy that females provided *less* direct care when breeding on larger carcasses, thus reducing their parental workload under favorable environmental conditions. This finding is indicative of load-lightening as reported in the cooperatively breeding superb fairy-wren, where females produce smaller eggs when anticipating favorable conditions due to presence of a larger number of helpers (Russell et al. 2007). Although females provided less direct care when breeding on larger carcasses, their larvae were nevertheless larger at dispersal. This finding might reflect that the larvae either have access to more food or are better at self-feeding directly from the carcass on larger carcasses.

We found evidence for differences in some trait values between females from the 2 source populations. Females from the Cornwall-derived population produced, on average, a larger clutch size and a larger number of dispersed larvae than females from the Edinburgh-derived population. Given that the beetles were reared in the laboratory under common environmental conditions, this finding suggests genetic differences between the 2 populations with respect to these traits. Thus, an avenue for future work is to investigate divergence in different measures of breeding success between multiple populations of the same species. Finally, we found significant differences in trait values between different batches, the implications of which we discuss below (see Methodological issues).

### Phenotypic correlations

We found evidence for both positive and negative associations across our range of traits, both between and within parental and offspring traits. Among parental traits, there were positive correlations between direct and indirect care, as well as between clutch size and indirect care. These positive phenotypic correlations suggest that associations between suites of parental traits are driven by variation in parental quality (i.e., resource acquisition) rather than by variation in how individuals allocate resources between different functions (i.e., trade-offs; van Noordwijk and de Jong 1986). If so, our results might reflect that higher quality females invest more effort in both prenatal investment in eggs and postnatal parental care. However, an alternative explanation is that females adjust the amount of direct and indirect care they provide in response to the number of offspring in the brood, which is positively correlated with clutch size (Table 2). For example, if some females invest more in egg production, they may have to increase their effort in postnatal care because they are caring for a larger brood. Further work is needed to examine whether these positive phenotypic correlations are driven directly by variation in female quality, or indirectly by females adjusting their level of posthatching care in response to brood size.

Our finding that there was a *positive* correlation between direct and indirect care differ from that in a previous study on the same species, which reported a strong *negative* phenotypic correlation between these traits (Walling et al. 2008). One potential explanation for this difference is the use of different methodology: Walling et al. (2008) collected behavioral data on direct, indirect, or no care over a relatively short timespan (i.e., 10min), whereas we measured the same behaviors over a longer timespan (i.e., 30min). Collecting behavioral data over a shorter timespan might generate a negative correlation between the 2 parental behaviors because many individuals would have engaged in only 1 of the 2 behaviors. In contrast, collecting such data over a longer timespan decreases this risk because a greater number of individuals would have engaged in all 3 behaviors (i.e., direct, indirect, and no care). Given that we also found positive correlations between clutch size and both direct and indirect care, our results suggest that phenotypic correlations between suites of parental traits are driven mainly by variation in parental quality rather than trade-offs in allocation between alternative traits.

Previous work on other taxa provides mixed evidence regarding the direction of any correlation between different components of parental care. For example, there is a positive correlation between provisioning and nest defence in female willow tits (Rytkönen et al. 1995), between nest sanitation and provisioning in barn swallows (Spencer 2005), and between incubation and food provisioning in house sparrows (Kopisch et al. 2005). Meanwhile, there is a negative correlation between amount of food provided per visit and nest defence in male willow tits (Rytkönen et al. 1995) and an increase in egg fanning is associated with a decrease in nest defence in male sand gobies (Lissåker and Kvarnemo 2006). In general, we would expect positive correlations between components of parental care when variation in parental quality exceeds variation due to trade-offs, whereas we would expect negative correlations when variation due to trade-offs exceeds variation in parental quality (van Noordwijk and de Jong 1986). Thus, the fact that there is evidence for both positive and negative correlations between different components of parental care suggests that there often are differences between species in the relative contributions of parental quality and trade-offs. In light of this, we suggest that a potential avenue for further work in this field is to examine how factors associated with parental quality, such as body condition, age, and territory quality, might alter the direction of any correlation between different components of parental care.

Our experimental design allows us to exclude some potential sources of variation underpinning the proposed variation in parental quality in *N. vespilloides*. First, we can exclude effects due to heterogeneity in the amount of resources available for breeding (i.e., carcass size), given that we provided parents with either a large or a small carcass and then controlled for effects of carcass size in our statistical analyses. Second, we can exclude effects due to heterogeneity in prior breeding experience because we always used inexperienced virgin females. Recent studies on this species have reported that large females lay larger eggs, provide more postnatal care and produced larger offspring than small females (Steiger 2013; Pilakouta et al. 2015). Thus, one potential source of variation in heterogeneity is female size, and future work should now examine how female size influences correlations between parental traits.

With respect to parental and offspring traits, we found a positive phenotypic correlation between time spent begging by larvae and the time females spent providing indirect care. We anticipated phenotypic correlations between parental and offspring behaviors due to parent–offspring communication whereby parents adjust their care decisions in response to offspring begging or vice versa. Theoretical and empirical work in this field suggests that offspring begging should be more strongly correlated with *direct* care, given that this form of care includes parental food provisioning (Godfray 1991; Smiseth and Moore 2002). It is less obvious that parent–offspring communication would cause a correlation between larval begging and *indirect* care, which is associated with defense against microbial competitors (Rozen et al. 2008). However, our results may reflect that females respond to an increase in larval begging by preparing the carcass in such a way that it reduces microbial competition and facilitates self-feeding by larvae. Thus, we suggest that multivariate studies might provide new insights by testing for correlations that otherwise would be ignored. An alternative explanation for the correlation between indirect care and larval begging is that larvae were hungrier when females spent more time providing indirect care due to a trade-off between indirect and direct care. We can exclude this explanation because we found evidence for a positive correlation between indirect and direct care. A final explanation is that higher quality females both provide more care and produce larvae that beg more. There is now a need for future studies to examine whether variation in female quality (e.g., due to female body size) might drive associations between parental and offspring traits.

We found that dispersal mass was positively correlated with both egg size and direct care, but negatively correlated with clutch size. Furthermore, the number of dispersing larvae was positively correlated with clutch size and indirect care. These findings provide evidence for phenotypic correlations between the 4 parental traits and our 2 measures of breeding success (i.e., dispersal mass and number of dispersing larvae), reflective of an integrated set of multiple aspects determining variation in parental care and juvenile development. The patterns of these correlations provide new insights into how parental traits may enhance different components of breeding success. For example, our results suggest that females might produce larger larvae by laying larger eggs and providing more direct care, whereas females might produce a larger number of larvae by laying more eggs and providing more indirect care. These interpretations seem reasonable from a functional point of view: Egg size and direct care are likely to be positively correlated with offspring size given that these traits are associated with access to food resources. Meanwhile, clutch size and indirect care are likely to be positively correlated with the number of offspring produced given that clutch size determines the upper limit to the number of offspring that can be produced, while indirect care (e.g., deposition of antimicrobials) might reduce offspring mortality due to competition from microbes (Rozen et al. 2008).

Finally, we found a negative phenotypic correlation between dispersal mass and number of dispersing larvae, indicative of the well-supported trade-off between the number and size of offspring (Smith and Fretwell 1974). Our finding is also consistent with previous work on this species (e.g., Rozen et al. 2008; Monteith et al. 2012; Smiseth et al. 2014). The trade-off is presumably driven by the nature of the system given that the size of the carcass determines the amount of resources that are available for breeding. Thus, even though higher quality females may lay more eggs and provide more direct and indirect care, the amount of resources would inevitably be limited by the size of the carcass upon which they breed. The finding that clutch size was positively correlated with number of dispersing larvae and negatively correlated with dispersal mass suggests that clutch size plays an important role as a mechanism determining variation among females in this trade-off. For example, females could produce a larger number of relatively small offspring by increasing the clutch size, or they could produce a smaller number of relatively large offspring by reducing the clutch size.

### Heritabilities and genetic correlations

We found evidence for significant additive genetic variance in clutch size and brood size (number of dispersed larvae; Table 3) after controlling for environmental effects, although the estimated heritabilities for these 3 traits were relatively low. We note that our 2 populations of origin also differed with respect to clutch size and brood size, suggesting genetic differences both between and within populations with respect to these 2 traits. The only traits that showed significant additive genetic variance in our analyses were a life-history trait (clutch size) and a measure of breeding success (number of dispersed larvae) that was closely linked to that life-history trait. In contrast, we found no significant additive genetic variance for any parental or offspring behaviors, suggesting that these traits would show a very limited response to selection. It had previously been suggested that behavioral traits in general may have lower heritabilities than morphological or life-history traits (Mousseau and Roff 1987), presumably reflecting high levels of phenotypic plasticity in behavioral traits. However, a recent review by Postma (2014) shows that average heritability estimates for behavioral traits were similar to those for morphological traits and higher than those for life-history traits.

We found evidence for a positive genetic correlation between clutch size and number of dispersing larvae, while there were no genetic correlations between different parental care traits or between parental and offspring behaviors. The positive genetic correlation between clutch size and number of dispersing larvae suggests that selection acting on any one of these traits would lead to a correlated response in the other trait such that they would not evolve independently. The limited evidence for significant genetic correlations between parental care traits in our study may reflect the low heritabilities of most traits. In a previous study on the same species, Walling et al. (2008) examined genetic correlations between parental traits within and between the 2 sexes. Within females, this study found a negative genetic correlation between direct and indirect care and between indirect care and number of dispersing larvae, and a positive genetic correlation between direct care and number of dispersing larvae. There are several possible explanations for the difference between these results and ours, including 1) differences in the methodology used to record parental behavior (see above), 2) differences in experimental design (Walling et al. estimated genetic correlations in the presence of the male partner while we did so in his absence), and 3) differences in statistical models (we removed potential confounding effects due to differences between batches; see Methodological issues below). It is not possible to distinguish between these explanations based on our data, but we urge other researchers to carefully consider these issues when designing further work in this field.

Finally, we found no evidence for genetic correlations between parental and offspring behaviors. Genetic correlations between offspring begging and parental food provisioning have been interpreted as evidence for coadaptation between parents and offspring (Kölliker et al. 2012). Previous studies on different species have reported genetic correlations between these 2 traits, although, critically, with variation in the direction of this genetic correlation. For example, a positive genetic correlation between offspring begging and parental food provisioning has been reported in great tits (Kölliker et al. 2000), mice (Hager and Johnstone 2003), and *N. vespilloides* (Lock et al. 2004), while a negative genetic correlation has been found in the seed bug *Sehirus cinctus* (Agrawal et al. 2001), rhesus macaques (Maestripieri 2004), and blue tits (Lucass et al. 2016). It is currently unclear why there is such variation in the direction of these genetic correlations, but one potential explanation is that this might indicate the relative strength of selection acting on parental and offspring traits (Kölliker et al. 2005). We also note that these studies are based on different experimental designs: some studies use a cross-fostering design whereas others do not, some studies measure behavioral reactions norms while others do not, and some studies measure begging toward a standardized stimulus and others do not. In a previous study on the same system, Lock et al. (2004) found a positive genetic correlation between larval begging and female food provisioning. Again, this study used very different experimental design to ours. Lock et al. (2004) used a cross-fostering experiment to test for a correlation between larval begging and parental food provisioning when larvae were begging to a foster parent and females were provisioning food to a foster brood. This design eliminates phenotypic effects due to behavioral adjustments between females and their offspring, but would not eliminate maternal effects on offspring begging mediated through the eggs. On the other hand, cross-fostering designs may have a greater statistical power because they can experimentally control for some confounding factors, such as variation in brood size.

### Methodological issues

Although we conducted our experiments under controlled laboratory conditions, where both temperature and light conditions were held constant, we found significant differences between batches for all traits, except time spent begging. We note that our laboratory populations had been kept in the lab for multiple generations (range: 2–13), thus making it unlikely that the difference batches were due to potential maternal or grand-maternal effects arising as a consequence of wild-caught individuals being collected at different times of the year. Although the source of the observed differences between batches remains unknown, our results highlight the importance of statistically controlling for confounding effects due to this cryptic source of environmental variation. Table 3 provides an overview of heritability estimates based on univariate models after controlling for potential effects due to batch. As stated earlier, the estimated heritabilities were relatively low or very low and statistically significant only for clutch size and number of dispersing larvae. When we excluded batch and population of origin from the models, there were significant heritabilities for 6 out of 7 traits (Table 3). Thus, had we not controlled for these effects, we would in many cases overestimate heritabilities more than 2-fold. The most likely reason for this is that, when batch is excluded from the models, any effects due to environmental heterogeneity between batches would now be attributed to additive genetic variation between different families used in the different batches. Thus, we suggest that future breeding experiments in this field include batch as a factor in statistical models rather than assuming that the constant laboratory conditions will exclude all variation in environmental conditions. Furthermore, when population of origin is excluded from the models, any effects due to genetic differences between populations would be attributed to additive genetic variation.

## CONCLUSIONS

Here, we have described phenotypic and genetic correlations between parental and offspring traits in the burying beetle *N. vespilloides*. Our study illustrates how information on the phenotypic and genetic correlations between parental and offspring traits can provide valuable new insights into the evolution of elaborate parental care. First, our results show how environmental conditions affect both parental care behaviors and breeding success. Second, we found that phenotypic correlations between parental traits were consistently positive, suggesting considerable variation in parental quality among female parents. Third, we found phenotypic correlations between parental traits and 2 indicators of breeding success (dispersal mass and number of dispersing larvae), suggesting that some such traits influence offspring size while others influence number of offspring that are produced. Fourth, we found that most parental and offspring traits have either relatively low or very low heritabilities, suggesting that these traits would respond slowly to selection. The only 2 traits to show significant additive genetic variation were clutch size and number of dispersing larvae, and these 2 traits were also the only 2 traits that differed between the 2 populations of origin. Finally, there were very few genetic correlations between traits, suggesting that there are few genetic constraints on the evolution of parental and offspring traits and that there is little evidence for coadaptation between parents and offspring. We encourage future work in this field to apply the multivariate approach used in our study to other systems with elaborate parental care. Such work could provide new insights into trade-offs between different parental traits, communication between parents and offspring, and potential genetic constraints on the evolution of parental and offspring traits.

## Funding

Funding was provided by Natural Environment Research Council (NE/G004293/1) (P.T.S. and L.E.B.K.) and Australian Research Council Future Fellowship (FT110100453) (L.E.B.K.).
